# Isolation and Evaluation of *Streptomyces melanogenes* YBS22 with Potential Application for Biocontrol of Rice Blast Disease

**DOI:** 10.3390/microorganisms11122988

**Published:** 2023-12-15

**Authors:** Luyang Song, Fei Wang, Chuang Liu, Zhengzhe Guan, Mengjiao Wang, Rongrong Zhong, Huijun Xi, Ying Zhao, Caiyi Wen

**Affiliations:** 1 College of Plant Protection, Henan Agricultural University, Zhengzhou 450046, China; lysong@henau.edu.cn (L.S.); 17638563265@163.com (C.L.); gzzhnnd7@163.com (Z.G.); w1225291093@163.com (M.W.); zhongrongrong2021@126.com (R.Z.); xihuijun1994@126.com (H.X.); 2 Institute of Plant Protection, Henan Academy of Agricultural Sciences, Zhengzhou 450002, China; yunfeiren@163.com

**Keywords:** *Streptomyces melanogenes* YBS22, antifungal activity, antimycin, *Magnaporthe oryzae*, rice blast

## Abstract

Plant diseases caused by pathogenic fungi pose a significant threat to agricultural production. This study reports on a strain YBS22 with broad-spectrum antifungal activity that was isolated and identified, and its active metabolites were purified and systematically studied. Based on a whole genome sequence analysis, the new strain YBS22 was identified as *Streptomyces melanogenes*. Furthermore, eight gene clusters were predicted in YBS22 that are responsible for the synthesis of bioactive secondary metabolites. These clusters have homologous sequences in the MIBiG database with a similarity of 100%. The antifungal effects of YBS22 and its crude extract were evaluated in vivo and vitro. Our findings revealed that treatment with the strain YBS22 and its crude extract significantly reduced the size of necrotic lesions caused by *Magnaporthe oryzae* on rice leaves. Further analysis led to the isolation and purification of an active compound from the crude extract of the strain YBS22, identified as *N*-formylantimycin acid methyl ester, an analog of antimycin, characterized by NMR and MS analyses. Consistently, the active compound can significantly inhibit the germination and development of *M. oryzae* spores in a manner that is both dose- and time-dependent. As a result, we propose that the strain YBS22 could serve as a novel source for the development of biological agents aimed at controlling rice blast disease.

## 1. Introduction

*Actinomycetes*, as a crucial microbial resource, have made significant contributions to the advancement of the biotechnology, medical, and agricultural sectors [1,2,3]. The genus *Streptomyces*, belonging to the Actinobacteria phylum, is known for its high sporulation capacity and the thick cell wall of their spores, which result in long-term survival [4]. The biological activity of a variety of metabolites produced by *Streptomyces* spp. Includes antitumor, antiviral, antihypertensive, antibiotic new drugs, immunosuppressive agents, biological enzymes, and the applications of the other biologically active substances have been extensively studied [5]. Meanwhile, *Streptomyces* can produce a variety of extracellular enzymes to degrade chitin, cellulose, and animal stumps in soil. Moreover, it also affects other soil microbial metabolism by producing protein biosynthesis inhibitors or regulators, which is considered as one of the most important biological factors to form a stable soil micro-ecological environment of agriculture [6,7,8,9]. In addition, *Streptomyces* also stimulate and enhance several direct and indirect biosynthetic pathways in plants. These include inorganic phosphate solubilization biosynthesis of chelating compounds, phytohormones production, inhibition of plant pathogens, and alleviation of various abiotic stresses [10]. Furthermore, certain strains of *Streptomyces* colonize plant roots, and they actively antagonize and compete with pathogenic microorganisms responsible for soil-borne plant diseases [11,12]. In total, *Streptomyces* holds great potential as a biocontrol agent due to its ability to inhibit a wide range of plant pathogens and promote plant growth.

Phytopathogenic fungi, increasingly recognized as a significant threat to global agricultural production, cause a variety of plant diseases. Among these fungi, *M. oryzae* stands out as the most destructive, earning it the top rank among fungal plant pathogens. This particular fungus is responsible for the devastating rice blast disease [13,14]. Currently, chemical fungicides are typically used to control the blast disease, including carpropamid, fenoxanil, tiadinil, probenazole, tricyclazole, azoxystrobin, isoprothiolane, and propiconazole [15]. However, overuse of chemical fungicides in agriculture has threatened human health and the environment, and it also leads to increased drug resistance of pathogens [16]. Therefore, an increasing demand for bio-pesticides or environment-friendly crop protection products has become a priority of the research and funding agencies all over the world. Antifungal activity has been reported in microorganisms, especially actinomycetes, and many reports have shown that actinomycetes can protect plants and show antagonism against several plant pathogenic fungi [17,18,19,20]. *Streptomyces endus* OsiSh-2 exhibited remarkable antagonistic activity to *M. oryzae* in vitro, and its spore solution (10^7^ spore mL^−1^) could reduce rice blast disease severity by 59.64% [21]. Previously, the *Streptomyces* strain UPMRS4 was selected as a potential biocontrol agent for rice blast disease because of its strong antagonistic activity against *Pyricularia oryzae* compared with other treatments [22]. This has led to the development of novel biofungicide formulations for rice blast disease using these *Streptomyces*. 

As the most common actinomycete in the environment, *Streptomyces* have made important contributions to the development of antibiotics [23]. The antimycin A family compounds are a class of natural antibiotics produced by *Streptomyces*, which possess potent antifungal, insecticidal, nematocidal, and anticancer activities [24]. A new antimycin-type antibiotic, kitamycin C was recently isolated from a culture broth of *Streptomyces antibioticus* strain 200–09. The compound exhibited strong antifungal activities against *Candida albicans* [25]. Two new antimycin A analogues were isolated from a spent broth of a marine-derived *Streptomyces lusitanus*, and these compounds also showed strong biological activity against *Staphylococcus aureus* and *Loktanella hongkongensis* [26]. The present study reports on the antimycin-producing biocontrol strain YBS22, which was identified via a whole genome sequence analysis, and the bioactive secondary metabolite gene clusters in the strain were predicted. The antifungal effects of YBS22 and its crude extract were evaluated in vivo and vitro. Moreover, the compound *N*-formylantimycic acid methyl ester, which belongs to an antimycin analog, was isolated and purified from crude extract produced by YBS22. We also investigated the antifungal activities of the analog against *M. oryzae*. The results from the present study revealed a new *Streptomyces* resource that could be utilized as a biological control agent on rice plants.

## 2. Materials and Methods

### 2.1. Isolation and Phenotypic Characteristics of the Strain YBS22

The strain YBS22 was isolated by the serial dilution method [27] from yellow-brown soil samples (sampling sites: 112°25′30″ E, 33°19′56″ N; pH 6.75; total nitrogen: 1.45%, total phosphorus: 0.11%, total potassium: 2.00%) in Henan Province, China. The antifungal activity of the isolate against these pathogenic fungi was examined by dual culture assay [28]. Culture features were investigated on the Gause’s synthetic agar medium No. 1. Morphological characteristics such as colony, aerial hyphae, substrate mycelium, and pigmentation were analyzed after 7–10 days of incubation at 28 °C. The mycelium morphology and spore chain of the YBS22 strain were visualized with scanning electron microscopy (SEM) (S-4800, Hitachi Limited, Tokyo, Japan) using a beam voltage of 5.0 kV.

### 2.2. Library Construction and Genome Sequencing

The genome was sequenced using a combination of Illumina sequencing platforms and Nanopore PromethION sequencing platforms. For Illumina sequencing, genomic DNA was used for each strain in sequencing library construction. DNA samples were sheared into 400–500 bp fragments using a Covaris M220 Focused Acoustic Shearer (Woburn, MA, USA) following the manufacture’s protocol. Illumina sequencing libraries were prepared from the sheared fragments using the NEXTFLEX Rapid DNA-Seq Kit. Briefly, the 5′ ends were first end-repaired and phosphorylated. Next, the 3′ ends were A-tailed and ligated to sequencing adapters. The third step was to enrich the adapters-ligated products using PCR. The prepared libraries then were used for paired-end Illumina sequencing (2 × 150 bp) on an Illumina Novaseq 6000 (Illumina Inc., San Diego, CA, USA).

For Nanopore sequencing, DNA fragments were repaired, purified, and then attached to the sequencing adapters supplied in the SQK-LSK109 kit to the DNA ends. Next, the Nanopore library was prepared and sequenced on Oxford Nanopore PromethION (Oxford Nanopore, Oxford, UK).

### 2.3. Genome Assembly and Annotation

The data generated from Nanopore and Illumina platform were used for bioinformatics analysis. All of the analyses were performed using the free online platform of Majorbio Cloud Platform (http://cloud.majorbio.com, accessed on 25 May 2022) from Shanghai Majorbio Bio-pharm Technology Co., Ltd. (Shanghai, China). The detailed procedures are as follows.

The raw Illumina sequencing reads generated from the paired-end library were subjected to quality-filtered using fastp v0.23.0. Nanopore reads were extracted, basecalled and demultiplexed, and trimmed with the minimum Q score cutoff of 7. Then, the clean short and long reads were assembled to construct complete genomes using Unicycle v0.4.8 [29]. As a final step, Unicycler used Pilon v1.22 to polish the assembly using short-read alignments, reducing the rate of small errors. The coding sequences (CDs) of chromosome and plasmid were predicted using Glimmer or Prodigal v2.6.3 [30] and GeneMarkS [31], respectively. tRNA-scan-SE (v 2.0) [32] was used for tRNA prediction and Barrnap v0.9 (https://github.com/tseemann/barrnap, accessed on 15 June 2023) was used for rRNA prediction. The predicted CDs were annotated from NR, Swiss-Prot, Pfam, GO, COG, KEGG, and CAZY database using sequence alignment tools such as BLAST, Diamond, and HMMER. Briefly, each set of query proteins was aligned with the databases, and annotations of best-matched subjects (*e*-value < 10^−5^) were obtained for gene annotation. The features of the chromosomes and plasmids were performed using BLAST Ring Image Generator (BRIG) [33]. KEGG and GO pathways were plotted by https://www.bioinformatics.com.cn (accessed on 10 November 2023), an online platform for data analysis and visualization. Biosynthetic gene clusters (BGCs) of secondary metabolites were identified by antiSMASH [34] (https://antismash.secondarymetabolites.org, accessed on 18 July 2023) with the default parameters.

### 2.4. Taxonomy of the Strain YBS22

The production of hydrogen sulfide (H_2_S), melanin, the liquefaction of gelatin, hydrolysis of starch, and cellulose, the utilization of carbon and nitrogen, the coagulation and peptonization of skim milk, a MR-VP test, a nitrate reduction test, the NaCl tolerance, and the ability to grow at different temperatures and pH were analyzed to determine the physiological and biological characteristics.

The genomic DNA was extracted from the antifungal strain YBS22 using the cetyltrimethylammonium bromide (CTAB) method. Partial sequences of the 16S rRNA gene were amplified using the primer set: forward primer 27F (5′-AGAGTTTGATCMTGGCTCAG-3′) and reverse primer 1492R (5′-TACGGYTACCTTGTTAYGACTT-3′). The polymerase chain reaction (PCR) was carried out at the thermal conditions of 35 cycles at 94 °C for 1 min, 59 °C for 30 s, and 72 °C for 1.5 min. The 16S rRNA gene was sequenced with the primer set (27F /1492R) by Sangon Biotech Co., Ltd. (Shanghai, China), and the SILVA was used as a reference for taxonomy assignment [35]. The 16S rRNA gene sequence was deposited in the NCBI GenBank database (Accession OR563648.1).

The sequence data were compared using the nucleotide basic local alignment search tool (BLAST) in GenBank. Multiple alignments of the sequence and phylogenetic analysis were performed using the MEGA X software, and the phylogenetic tree was constructed using the maximum likelihood (ML) method with 1000 replicates [36]. The average nucleotide identity (ANI) and average amino acid identity (AAI) were calculated using the FastANI algorithm. The genomes of the bacterial strains used for AAI and ANI calculations include *Streptomyces rubiginosohelvolus* JCM 4415 (BMTW01000001); *Streptomyces yangpuensis* CM253 (CP102514); *Streptomyces flavotricini NGL1* (JAINUL010000001); *Streptomyces melanogenes* JCM 4398 (BMTS01000001); *Streptomyces badius* JCM 4350 (BMSZ01000001); *Streptomyces anulatus* YINM00001 (CP086102); *Streptomyces amritsarensis* MTCC 11845 (MQUR01000012); *Streptomyces pluricolorescens* JCM 4602 (JAINUL010000001); *Streptomyces albovinaceus* NRRL B-2566 (MUAX01000001); and *Streptomyces tanashiensis* Kala (CP084204). An ANI value cutoff of 96% is recommended for species delineation [37].

### 2.5. Antifungal Activity of the Strain YBS22 and Its Active Compounds

Using the agar disk diffusion method, the strain YBS22 was evaluated for its in vitro potential to inhibit several animal and plant pathogens, including *M. oryzae*, *Fusarium oxysporum* f. sp. *Momodicase*, *Botryosphaeria dothidea*, *Collectotrichum orbiculare*, *Exserohilum turcicum*, *Gaeumannomyces graminis* var. *tritici*, *Fusarium graminearum*, and *Fusarium pseudograminearum*. For the fungal pathogen strains, a block of mycelium with a 6 mm-diameter was placed onto the center of a sterile potato dextrose agar (PDA) plate, and then cultures of the strain YBS22 were streaked with sterilized toothpicks at distance of 2.5 cm away from the margins of the mycelia colony and cultivated at 28 °C in darkness for 7–10 days. The PDA plate inoculated only with the pathogenic fungi was used as the control.

The diameter of the Inhibition zones and the antifungal index of mycelial growth of YBS22 were measured following the methods of Wang et al. [38].

Regarding the antifungal activity of crude extract, a small amount of crude extract was dissolved in methanol. It was configured to 10 mg/mL as a stock solution and the mother liquor was diluted to 200 μg/mL with sterile water. The samples were diluted from stock solution to 200 μg/mL with sterile water and filtered through a sterile 0.22 μm filter. We measured the antifungal activity of the crude extract by a filter paper method.

For the in vitro pathogenicity test on rice leaves, the rice seeds were surface sterilized and incubated in a petri dish lined with filter paper at 30 °C for a period of three days. Following this, the seeds were transplanted to a greenhouse. Once the rice seedlings reached the four-leaf stage, leaves from the same position on each seedling were selected. These leaves were cut into sections measuring 5 cm in length and subsequently rinsed with sterile water. The leaf sections were then categorized into four groups for further treatments; each of the following represents a different treatment: (a) Control: treatment with sterile water; (b) Treatment with sterile water after inoculation with 10 μL *M. oryzae* spores (inoculated with 3 × 10^5^ spores/mL containing 0.05% Tween-20); (c) Treatment with 200 μg/mL crude extract of YBS22 after inoculation with *M. oryzae*; (d) Treatment with the culture supernatant of YBS22 after inoculation with *M. oryzae*. Each treatment was replicated five times.

### 2.6. Extraction, Purification, and Identification of the Antifungal Active Compounds

The PDA medium was employed for the preliminary seed culture. The antifungal strain YBS22 was inoculated onto Gause’s synthetic agar medium No. 1 and incubated at a temperature of 28 °C for a duration of 7 days. Subsequently, five spore cakes, each with a diameter of 6 mm, were collected and transferred into a 250 mL flask that contained 100 mL of the seed medium. This culture was then maintained at a constant speed of 180 rpm for a period of 72 h at a temperature of 28 °C.

The solid-state fermentation medium was composed of corn bran (300 g/kg), wheat bran (700 g/kg), CaCO_3_ (20 g/kg), and H_2_O (450 mL/kg), with the pH adjusted to 8.0. This medium was allocated across 40 sets of 0.5 L beakers and sterilized at 121 °C for an hour in an autoclave. Post-sterilization, seed cultures prepared aseptically were inoculated into the medium to achieve a final concentration of 10%. The cultures were incubated for a period of 30 days at 28 °C. Following incubation, the fermented material underwent extraction with a methanol-ethyl acetate mixture (ratio = 1:2) for a duration of 24 h. The extraction supernatant was left undisturbed overnight and subsequently filtered through filter paper to eliminate any precipitate. The filtrate was then evaporated under reduced pressure to obtain the crude extract.

The crude extracts were subjected to flash chromatographic separation (8 cm × 70 cm column) with 200–300 mesh silica gel (Qingdao Marine Chemical Factory, Qingdao, China) as the stationary phase. Fraction T2 was then subjected to silica gel chromatography (6 cm × 40 cm column) using dichloromethane/methanol (100:1), and five fractions (marked as T2-1 to T2-5) were obtained. The resulting Fraction T2-2 was separated by Sephadex LH-20 column chromatography (Sephadex LH-20, Beijing Solarbio Science & Technology Co. Ltd., Beijing, China; 1.5 cm × 110 cm column; flow rate: 1 mL/min) with dichloromethane/methanol (1:1) as the eluent, and three subfractions (marked as T2-2-1 to T2-2-3) were collected. Subfraction T2-2-2 was purified by recycling preparative high-performance liquid chromatography (HPLC, LC-9101, Japan Analytical Industry Co., Ltd., Tokyo, Japan) using a Shim-Pack column (JAIGEL-ODS-AP, 250 mm × 20 mm, 15 μm) with methanol/water (70:30) as mobile phase and 3.5 mL/min as the flow rate. The eluents with retention time between 49.0 and 52.0 min was collected, which was thereafter identified as the analog of antimycin, *N*-formylantimycic acid methyl ester, marked as compound **1**. The HPLC detection was performed on an Agilent 1200 series HPLC system (Agilent Technologies, Santa Clara, CA, USA, Agilent TC-C18, 250 mm × 4.6 mm, 5 μm), G1314B photodiode array detector at 254 nm. The purity of the products was determined by thin-layer chromatography (TLC) and spots were visualized under a UV lamp at 254 nm and stained with phosphomolybdic acid (PMA). The nuclear magnetic resonance (NMR) spectra were recorded on a Varian INOVE-500 spectrometer (Varian Associates Inc., Palo Alto, CA, USA) operating at 500 MHz for ^1^H and 125 MHz for ^13^C and using tetramethylsilane (TMS) as an internal standard. The high-resolution electrospray ionization mass spectroscopy (HRESIMS) data were recorded on a LCMS-IT-TOF system (Shimadzu, Kyoto, Japan) incorporating a Prominence UFLC system and an ESI interface.

### 2.7. Conidial Germination Inhibition Assays

*M. oryzae* conidia was prepared as described by Sugihiro [39]. The conidia were diluted with sterile water up to 1 × 10^5^ spore/mL. Compound **1** was dissolved with methanol (10 mg/mL) and then diluted to 100, 50, 10 μg/mL with sterile water, respectively. An equal volume of the spore suspension and compound **1** at different concentration was mixed and a final concentration was configured at 50, 25, and 5 μg/mL. Equal volume of sterile water in the mixture with spore suspension served as a control. About 60 μL of the mixed solution was dropped on a clean and sterile concave glass slide, placed onto a petri dish covered with wet filter paper, and the culture was moisturized at 28 °C. The spore germination status was observed under optical microscopy at 4 and 8 h, and spore germination rate and germ tube length were recorded and calculated following the formula below. The test was repeated for three times.

Spore germination rate = Number of spore germinated/Total number of spores × 100%

### 2.8. Statistical Analysis

All data were presented as the mean ± standard deviation (SD). One-way analysis of variance (ANOVA) was employed to test for significant differences (*p* < 0.05) using SPSS Statistics 23 (IBM Corp., Armonk, NY, USA).

## 3. Results

### 3.1. Identification and Phenotypic Characteristics of the Strain YBS22

**Figure 1:**
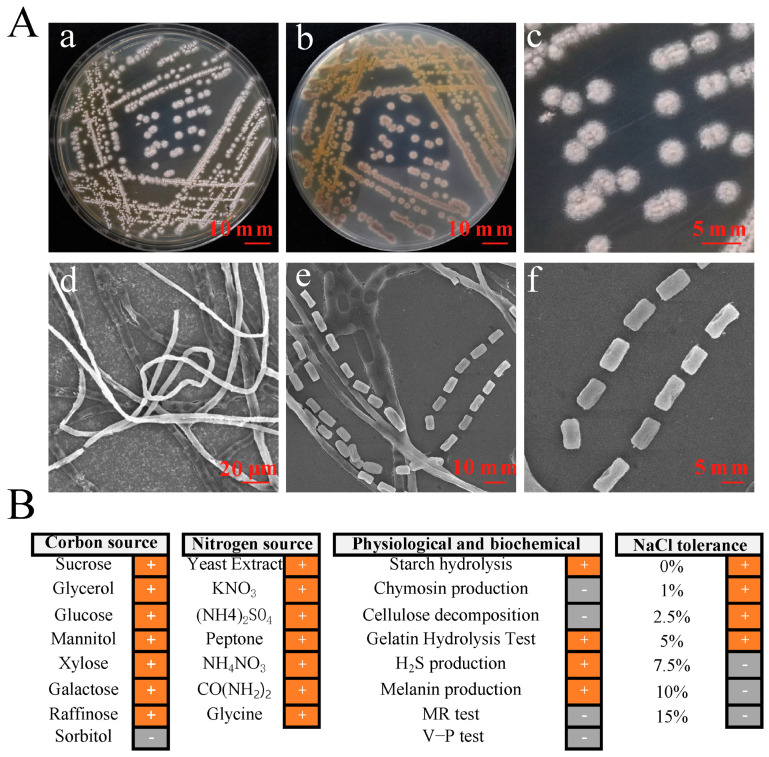
Phenotypic characteristics and biochemical properties of the strain YBS22. (**A**); Characteristics and micromorphology of the strain YBS22 on Gause’s medium. (**a**) Front. (**b**) Back. (**c**) Single colony. (**d**) Aerial hyphae (SEM3500×). (**e**) Spore producing chains (SEM 6000×). (**f**) Spores (SEM 6000×). (**B**); Physiological and biochemical characteristics of the strain YBS22. “+” indicates positive reaction, “-” indicates negative reaction.

A total of 32 actinomycetes strains were isolated from yellow-brown type soil. They were screened for their antifungal activity against 9 types of pathogenic fungi. Among them, only the strain YBS22 has the strongest antagonistic activity against *M. oryzae* (Figure S1). The strain YBS22 exhibited a white color and a round, powdery morphology, which was difficult to fully induce on Gause’s synthetic agar medium No. 1 (Figure 1(Aa–c)). Electron microscope imaging of the aerial hyphae of the strain YBS22 revealed a straight morphology with few branches, measuring 4–6 μm in diameter (Figure 1(Ad)). The aerial mycelia gave rise to spore filaments, and septa are formed to create cylindrical spores with dimensions of 8–12 μm in length and 4–6 μm in width (Figure 1(Ae,f)). 

Among 31 phenotypic items tested, the strain YBS22 showed positive reactions in the 22 items including xylose utilization, melanin production, galactose utilization, H_2_S production, cellulose decomposition, the gelatin hydrolysis test, utilization of seven nitrogen sources (yeast extract, potassium nitrate, ammonium nitrate, ammonium sulfate, peptone, urea and glycine), and growth on LB agar plate with 1–5% of NaCl. Negative reactions were confirmed in the 9 items including methyl red (MR) and Voges-Proskauer (V-P) tests, sorbitol utilization, chymosin production, cellulose decomposition, and growth in 7.5–15% of NaCl (Figure 1B).

**Figure 2:**
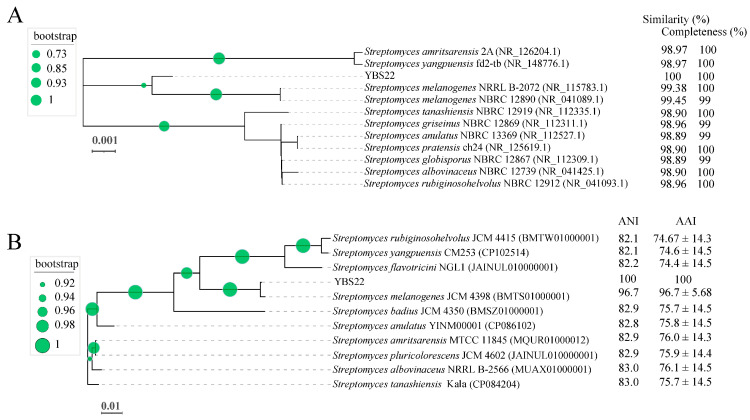
Phylogenetic analysis. (**A**); Phylogenetic tree based on 16S rRNA sequences obtained by the maximum likelihood (ML) method with 1000 replicates. (**B**); A ML phylogenetic tree was constructed using selected bacterial genome sequences. The values next to the tree indicate ANI and AAI values. Circle at nodes indicate the levels of bootstrap values (1000 replicates).

The nearly full-length 16S rRNA gene nucleotide sequencing (1490 bp) of the strain YBS22 was amplified and subjected to a similarity-based blast against the taxonomically united 16S rRNA database in NCBI. Blast results showed 99.45%, 99.38%, 98.96%, and 98.90% similarities with the corresponding genes from *S. melanogenes* NBRC 12980, *S. melanogenes* NRRLB 2072, *S. griseinus* NBRC 12869, and *S. tanashiensis* NBRC 12919, respectively. Phylogenetic tree with the maximum likelihood (ML) showed the strain YBS22 with high similarity to the type strains of the *S. melanogenes* (Figure 2A). To further evaluate its taxonomic position within the *Streptomyces* genus, the complete genome of YBS22 was sequenced. Additionally, 11 public type reference genomes of closely related *Streptomyces* species were collected for subsequent analysis. In the core genome tree, YBS22, together with *S. melanogenes* JCM4398, formed a monophyletic clade, which was deeply nested within the genus *Streptomyces*. The YBS22 chromosome shared 96.7% average nucleotide identity (ANI) and 96.7 ± 5.68% average amino acid identity (AAI) with *S. melanogenes* JCM4398 (Figure 2B). The ANI values of 96.7% exceeded the recommended cutoff of 96% for species delineation. Based on the above results, it is suggested that the strain YBS22 was a member of *S. melanogenes* species.

### 3.2. Genomic Characteristics of YBS22

**Figure 3:**
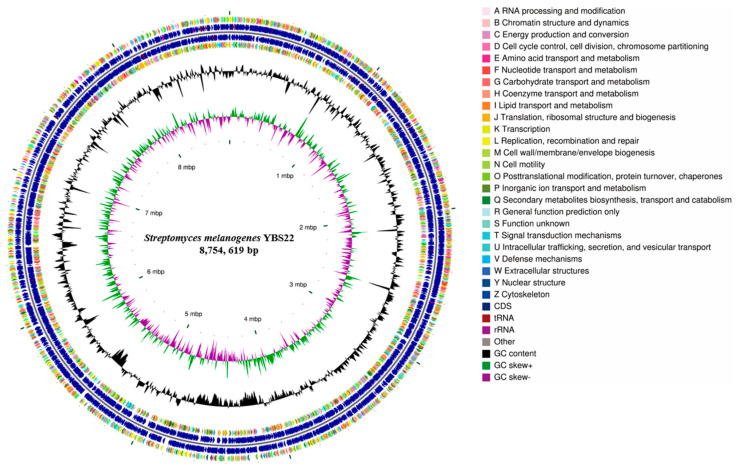
Genome features of the *S. melanogenes* YBS22 chromosome. Rings represent the following features labeled from inside to outside: ring 1, scale; ring 2, GC skew, green, and purple correspond to above- and below-average GC skew, respectively; rings 3, GC content; ring 4 and ring 7 represent the COG to which each CDS belongs; rings 5 to 6, blocks correspond to the positions of CDS, tRNA, and rRNA on the genome.

The complete genome of YBS22 consists of one single circular chromosome and four plasmids and was deposited in the NCBI GenBank database (Accession PRJNA1017970). The chromosome of YBS22 is approximately 8.7 million base pairs, with an average GC content of 71.6% (Figure 3; Table S1). In total, 8025 genes were predicted, including 7937 coding sequences (CDSs), 67 transfer RNA (tRNA), and 21 ribosomal RNA (rRNA) genes. Out of all the CDSs, 83.9% (6660 CDSs) were classified into COG (Clusters of Orthologous Genes; COG) families, which are composed of 24 categories (Figure 3). The results revealed that “E: Amino acid transport and metabolism” (509 genes), “G: Carbohydrate transport and metabolism” (553 genes), and “K: transcription” (855 genes) were the most enriched functional categories in the YBS22 chromosome. Only a small proportion of CDSs (10.9%) was poorly characterized (“R: General function prediction only”: 685 genes; “S: Functional unknown”: 183 genes).

According to GO annotation, a total of 33 GO terms were identified, approximately 895 genes are involved in biological processes, 1849 genes are involved in cellular components, while molecular function has the highest abundance with a total of 2121 genes (Figure S2, Table S2). According to KEGG annotation, 5850 genes were assigned to 32 KEGG pathways, which is 73.7% of all CDSs. Among these pathways, “metabolism” was the most represented with 2168 genes, followed by “Genetic Information Procession” and “Environmental Information Processing” pathways (Figure S3, Table S3).

### 3.3. Genes/Gene Clusters for Antibiotic Synthesis

The gene clusters related to secondary metabolite synthesis in the strain YBS22 were analyzed using the anti-SMASH method. In the genome of the strain YBS22, 39 putative biosynthesis gene clusters (BGCs) were discovered. Among them, 13 gene clusters have homologous sequences in the MIBiG database with a similarity of over 50%. It is most noticeable that 8 gene clusters with a homology sequence have a similarity of 100%, including NI-siderophore (BGC-3), ectoine (BGC-8), terpene (BGC-12), T3PKS (BGC-14), T1PKS (BGC-15), amglyccycl (BGC-16), lanthipeptide-class-iv (BGC-17), and NRPS-like (BGC-26) (Table S4).

**Figure 4:**
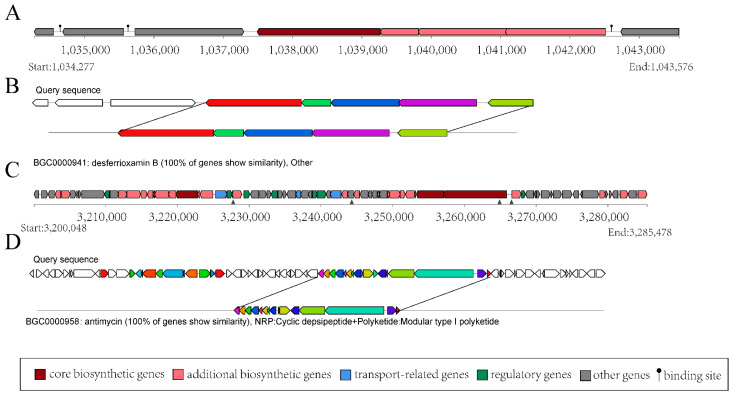
Gene clusters for secondary metabolism in the YBS22 chromosome. (**A**,**B**); Gene cluster structure analysis of the predicted compound desferrioxamin (**B**). (**C**,**D**); Gene cluster structure analysis of the predicted compound antimycin.

The structural analysis of BGC-3 is shown in Figure 4A, which comprises 8 coding sequences spanning a total length of 9299 base pairs (bp). Within BGC-3, 5 coding sequences correspond to the structural domains of *S. ficellus* NRRL 8067. The products encoded by these sequences bear a striking 100% similarity to desferrioxamin B (DFOB) (Figure 4B), which is a naturally occurring siderophore, originally identified in the soil-dwelling bacterium, *Streptomyces*. Type I polyketide synthases (T1PKSs) are one of the most extensively studied PKSs, and they play a key role in the biosynthesis of polyketide natural products, such as antibiotics. BGC-15 is predicted to be related to T1PKS biosynthesis in the genome of the strain YBS22. BGC-15 has a total length of 85,430 bp (Figure 4C). Within BGC-15, there are 15 coding sequences that are 100% similar to antimycin (Figure 4D), which has antifungal properties. These findings indicate that the strain YBS22 presents strong antagonistic activities.

### 3.4. Biocontrol Potential of the Strain YBS22

**Figure 5:**
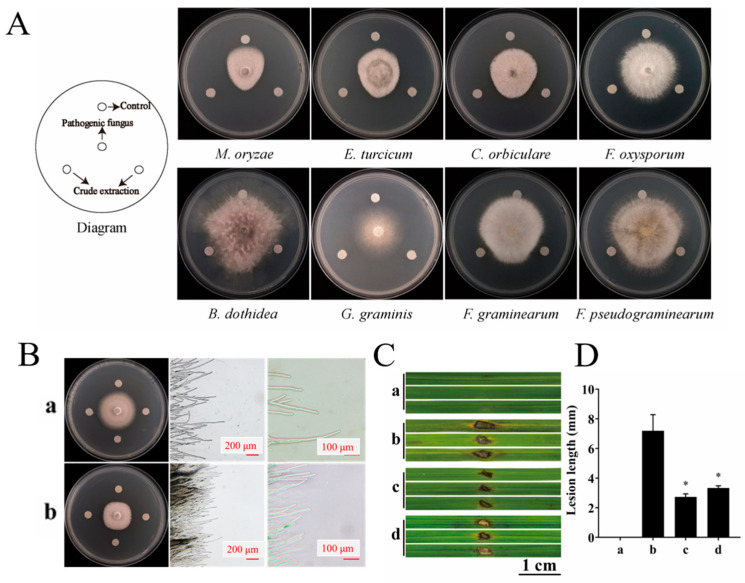
The antifungal effects of YBS22 and its crude extract were evaluated in vivo and vitro. (**A**); Antifungal activity of crude extract of the strain YBS22. (**B**); Antifungal activity of crude extract against *M. oryzae* and mycelial morphology. (**a**) Control group treated with methanol solvent; (**b**) Treatment with 200 μg/mL crude extract. (**C**,**D**); The length of lesions in different treatment. (**a**) Control: sterile water treatment; (**b**) Treatment with sterile water after *M. oryzae* inoculated; (**c**) Treatment with 200 μg/mL crude extract of YBS22 after *M. oryzae* inoculated, * ANOVA, *p* = 0.017; (**d**) Treatment with culture supernatant of YBS22 after *M. oryzae* inoculated, * ANOVA, *p* = 0.019. Significant different at the *p* < 0.05 level.

The dual culture assay revealed that the mycelial growth of all six fungal strains was inhibited to varying degrees by YBS22, showing broad-spectrum antifungal activity. Among them, it has the most obvious inhibitory zone and the strongest antagonistic effect against *M. oryzae* (Figure S4). To further evaluate the inhibitory ability of crude extracts produced by YBS22, the inhibitory zone was measured on eight plant pathogens using the filter paper method (Figure 5A). The crude extract of the strain YBS22 demonstrated inhibitory activity against all tested phytopathogens. It was most effective against *M. oryzae*, *Exserohilum turcicum*, and *Colletotrichum orbiculare*, with inhibition zones measuring 15.3 mm, 10.8 mm, and 10.1 mm in diameter, respectively. As can be seen from Figure 5B, in the control, rice blast fungus hyphae adhere to the culture medium surface, with sparse tips and normal branching. After treatment, a clear inhibition zone appears, many hyphae dissolve, bundle together, and lose their branches. A large number of rice blast fungus hyphae dissolve, the hyphae are bundled and not dispersed, and there is no branching.

To determine whether the antifungal activity was pH-dependent, we examined a range of pH values. The findings revealed that the efficacy of the crude extract was indeed influenced by pH values. Optimal activity was observed at a neutral pH of 7.0, which was significantly higher than that seen in the more acidic range of pH 3–5. Conversely, alkaline conditions (pH 9.0–11.0) had a detrimental effect on the antifungal activity (*p* < 0.05). In addition, the crude extract of the strain YBS22 possessed good thermal stability at temperatures below 60 °C. Moreover, it still exhibited bioactivity against *M. oryzae* upon treatment at 121 °C for 30 min (Table S5). 

Based on the above results, we deduced that the crude extract of YBS22 has the greatest potential to suppress the rice leaf blast. To verify this hypothesis, we conducted a wounding inoculation experiment of detached rice leaves. The results indicated that the crude extract of the strain YBS22 could significantly inhibit the development of necrotic lesions (Figure 5Cc,d). When treated with the crude extract or culture supernatant of the strain YBS22 after inoculation of *M. oryzae*, a lower index of the disease was observed and the average lengths of the lesions were 2.73 ± 0.21 mm and 3.33 ± 0.22 mm compared with the control treatment, respectively (Figure 5D) (*p* < 0.05). To further investigate the active components in the crude extract, a gradient elution method was used with the mixture of methanol and dichloromethane (100:0–0:100) as the eluent. Eight fractions were collected and marked as fraction T1-T8 according to their eluting order, respectively. Among them, T2 has the strongest inhibitory activity against rice blast fungus (79.32% ± 5.47%) (Figure S5A, Table S6). Subsequently, using secondary silica gel column chromatography, gel column separation, and dual culture assay methods, it was ultimately determined that component T2-2-2 was the main substance exerting antifungal activity (Figure S5B,C).

### 3.5. Preliminary Characterization of the Active Metabolites of YBS22

In order to clarify the structure of the antifungal agent T2-2-2 produced by YBS22, the T2-2-2 was purified using high-performance liquid chromatography (HPLC) with a Shim-Pack column (Figure S6). The eluent with retention time between 49.0 and 52.0 min was collected and dried as a yellow amorphous solid. Figure S7 shows the HPLC (TC-C18 column, 4.6 mm × 250 mm, 5 μm) chromatographic spectrum of the yellow amorphous solid, which displayed a single peak at about 3.09 min and marked as compound **1**. The molecular formula of the activate compound was subsequently confirmed as C_13_H_16_O_6_N_2_ by positive-ion high-resolution electrospray ionization mass spectrometry (HRESIMS) (*m*/*z* 319.0885 [M + Na]^+^, Figure S8). The ^1^H and ^13^C NMR spectra of compound **1** are shown in Figure S9A,B and Table S7. Supported by the data from mass spectrometry (MS), ^1^H NMR, and ^13^C NMR spectra, it can be concluded that compound **1** is derived from an analog of antimycin. This compound bears resemblance to an acyclic derivative of antimycin and has been identified as *N*-formylantimycic acid methyl ester (Figure S9C) [40]. It has been reported that *N*-formylantimycic acid methyl ester was isolated only from marine-derived *Streptomyces* and no other source [40,41].

### 3.6. Inhibitory Activity of Compound **1** on Spore Germination and Development of M. oryzae

**Figure 6:**
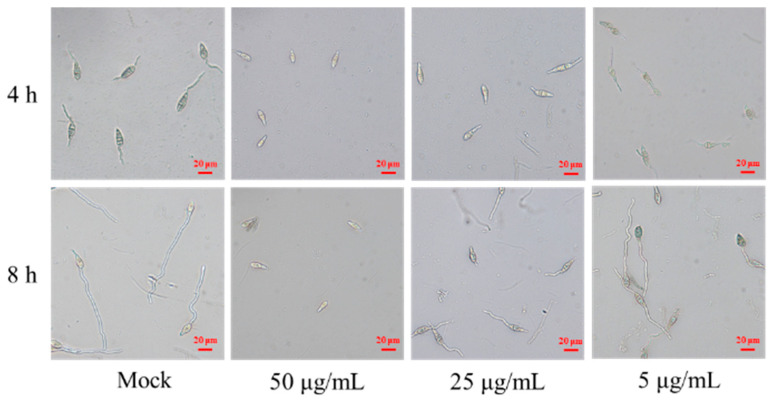
Conidial germination inhibition assays of compound **1**. Note: Mock: Sterile water control group; Treatment 1: Compound 1 is 50 μg/mL; Treatment 2: Compound **1** is 25 μg/mL; Treatment 3: Compound **1** is 5 μg/mL; Scale: 20 μm.

Next, the effect of the antimycin analog on *M. oryzae* spore was evaluated using conidia germination inhibition assays. We found that the antimycin analog markedly inhibited spore germination and development of *M. oryzae* in a dose-dependent manner after co-cultivation for 4 h (Table 1). When compared to the control group, which was treated with sterile water and had a germ tube length of 51.5 ± 6.5 μm, the inhibition rates for spore germination were found to be 21.57% and 100% upon treatment with compound **1** at concentrations of 5 μg/mL and 50 μg/mL, respectively. Correspondingly, the lengths of the germ tubes were reduced to 42.7 ± 4.7 μm and 0 μm at these same concentrations of compound **1** (Table 1). Moreover, we found that the spore germination inhibition rates were 0 and 100% upon treatment with compound **1** at 5 μg/mL and 50 μg/mL for 8 h, respectively (Figure 6, Table 1). The length of the germ tube was 73.8 ± 8.8 and 0 μm upon treatment with compound **1** at 5 and 50 μg/mL, respectively, which is significantly lower than the control (107.8 ± 8.8 μm). These results indicate that compound **1**, purified from *S. melanogenes* YBS22, can significantly inhibit the germination and development of *M. oryzae* spores in a manner that is both dose- and time-dependent.

## 4. Discussion

In the past few decades, research on *S. melanogenes* was very limited and focused on the production of melanin [42,43,44]. It has been reported that melanogenic actinomycetes have antagonistic activity against *Xanthomonas oryzae* [45]. Our results showed that the *S. melanogenes* strain YBS22 isolated from soil in Henan Province of China provided a broad spectrum of antifungal activities against plant pathogens, especially *M. oryzae*. Through fermentations and extraction, we obtained the crude extract of the strain YBS22. The crude extract also showed a broad-spectrum antifungal activity against six test phytopathogens; in addition, it had relatively stable inhibitory activity against *M. oryzae* at pH 7.0 and showed good thermal stability at temperatures below 60 °C (Table S5). There is evidence that pH and temperature stability of *Streptomyces* metabolites may provide a wider range of applications for agricultural production [46]. Thus, the strain YBS22 has a potentially therapeutic effect for plant diseases, and its antifungal activity makes it an excellent candidate for biological control of the rice blast.

Several studies have indicated that the presence of multiple copies of 16S rRNA has minimal impact on the phylogenetic analysis of species. This is because, in most instances, the sequences of these multiple copies are either identical or nearly identical [47,48,49]. Only the sequencing of the 16S rRNA conserved gene is insufficient to distinguish closely related species of *Streptomycetes*, due to the close correlations between these species [50]. Nonetheless, the enhanced accessibility of complete genome sequences has significantly aided in distinguishing these species. The ANI and AAI values, based on chromosome genome sequences, was computed. A cutoff of 96% was suggested for the delineation of species [51]. In the present study, the strain YBS22 displayed a 96.7% similarity to the *S. melanogenes* strain JCM4398, which is well above of the recommended threshold of 96% for species delineation. Thus, YBS22 was identified as *Streptomyces melanogenes.*

Products derived from *Streptomyces* spp. have been successfully formulated and tested for the control of various plant diseases. For instance, the primary activate compounds 4-methoxystyrene generated by *S. albulus* NJZJSA2 can inhibited mycelial growth by 56.3% [52]. The functions of the cell-wall degrading enzymes, β-1,3-glucanase, and chitinase, in contributing to the antifungal activity of *Streptomyces* species, have been extensively researched. Specifically, *S*. *angustmyceticus* NR8-2 is capable of producing cell-wall-degrading enzymes (such as β-1,3-glucanase), antifungal metabolites, and volatile antifungal compounds. These products play a significant role in its antifungal activity, inhibiting the growth of *Colletotrichum* sp. and *Curvularia lunata* by 75.6% and 69.5%, respectively [53]. 1*H*-pyrrole-2-carboxylic acid (PCA) was separated from *S. griseus H7602* and showed strong antifungal activity, and the mycelial growth of *Phytophthora capsici* was almost inhibited at a concentration of 64 μg/mL [54]. Compared to the active substance PCA produced by *S. griseus* H7602, the active compound (*N*-formylantimycin acid methyl ester) produced by the *S. melanogenes* strain YBS22 can completely inhibit the germination of *M. oryzae* spores at a concentration of 50 μg/mL (Figure 6, Table 1). This suggests that the antifungal activity of the strain YBS22 studied in this research has a higher potency and efficiency.

*Actinomycetes* produce a diverse array of biologically active metabolites, accounting for approximately 50% of the bioactive secondary metabolites discovered to date, including antibiotics, anticancer agents, anti-inflammatory substances, and enzymes [55]. In fact, *Streptomycetes* have been the source of many new antibiotic drugs, surpassing other bacteria and fungi [56]. The discovery of new antibiotics continues, with examples such as mediomycins A, B, and clethramycin from *Streptomyces* species showing broad antifungal activity [57]. Polyketides, important for their pharmaceutical applications, include erythromycin (antibacterial), nystatin (antifungal) [58], and avermectin (antiparasitic), all produced by *Streptomyces* species. Nonribosomal peptide synthetases (NRPSs) and type I polyketide synthases (PKS-I) are key players in the synthesis of a diverse range of significant bioactive compounds. These compounds, including antibiotics and siderophores, are produced by actinomycetes. These secondary metabolites hold considerable pharmacological importance, including their antifungal properties [59]. Our predicted results showed that YBS22 could coproduce three types of NPRS, including T3PKS (BGC-14), T1PKS (BGC-15), and NRPS-like (BGC-26) (Table S4), which may also contribute to its ability to antifungal activity. Siderophores produced by *Streptomyces* play a crucial role in promoting plant growth and improving plant tolerance to biotic and abiotic stress responses [60]. In the present study, based on genomic predictions, it is indicated that YBS22 has a 100% similarity with the biosynthetic gene clusters (BGCs) that produce siderophores (Figure 4A). Whether YBS22 also produces siderophores and whether these siderophores exert a growth-promoting effect on host plants are topics that remain to be further studied.

Previously, Seo, et al. isolated and identified a compound as *N*-formylantimycic acid methyl ester from *Streptomyces* sp. M03033 collected from marine sediment [40]. The evidence showed that the compound is a natural product with a similar structure and activity of antimycin (Figure S9C). These compounds in the antimycin group demonstrated their antifungal properties, and their biological activity was mainly affected by the acetylation on the C-8 position. In addition, it has been reported that there was an inverse relationship between the antifungal activity and the length of the 7-alkyl and 8-O-acyl side chains, which indicated that the antifungal potency of antimycins differed on their chemical structure [61]. Moreover, kitamycin A, kitamycin B, urauchimycin A, and urauchimycin B showed a lower biological activity due to the free hydroxyl group at C-8, and it was inferred that C-8 hydroxyl acylation enhanced the antifungal activity of the antimycin [62]. In our study, compound **1** exhibited a unique structure due to its D-configuration, which is identical to that of the threonine unit of *N*-formylantimycic acid methyl ester in antimycin. We identified this compound as an antimycin analog. In a previous study, *N*-formylantimycic acid methyl ester was inactive at any concentrations [40]. In the present study, compound **1** demonstrated potent biological activity against *M. oryzae* at a concentration of 50 μg/mL. We hypothesize that the biological activity of compound **1** is not associated with the function of the dilactone ring. Instead, the antifungal activity of this compound may be primarily driven by the formamide and phenolic hydroxyl groups. The presence of threonine does not appear to significantly contribute to its bioactivity [63]. Therefore, it is plausible that threonine could be substituted to enhance the biological activity of the antimycin analog and expand its antifungal spectrum. Further research is warranted to elucidate the inhibitory mechanism of *N*-formylantimycic acid methyl ester on *M. oryzae* and to investigate the influence of chemical structures on antifungal activity.

In conclusion, the *S. melanogenes* strain YBS22 exhibits significant potential as a biocontrol agent against rice blast disease. Its value for further development and utilization is considerable.

## 5. Conclusions

In conclusion, the strain *S. melanogenes* YBS22, along with its active compound **1** (*N*-formylantimycin acid methyl ester), could serve as novel sources of potential biocontrol agents against various plant pathogens. This is particularly relevant for the rice blast pathogen *M. oryzae*.

## Figures and Tables

**Table 1:** Conidial Germination Inhibition Assays of Compound **1** ^a^.

Treatment	4 h		8 h	
	Germination Rate (%)	Germ Tube Length (μm)	Germination Rate (%)	Germ Tube Length (μm)
Mock	90.00	51.5 ± 6.5 a	100.00	107.8 ± 8.8 a
50 μg/mL	0.00	-	0.00	-
25 μg/mL	9.76	6.8 ± 2.2 b	68.42	47.2 ± 12.2 c
5 μg/mL	70.59	42.7 ± 4.7 c	100.00	73.8 ± 8.8 d

^a^ Treatment: Treated with different concentration of compound **1**; Mock: Equal volume of sterile water in the mixture with spore suspension served as a control. Data were presented as mean ± SD, different letters indicate significantly different groups (*p* < 0.05, ANOVA, Tukey HSD).

## Data Availability

The complete genome of YBS22 consists of one single circular chromosome and four plasmids and was deposited in the NCBI GenBank database (Accession PRJNA1017970).

## Supplementary Materials

The following supporting information can be downloaded at: https://www.mdpi.com/article/10.3390/microorganisms11122988/s1, Figure S1: Antagonistic activity profiles of the typical strains; Figure S2: GO annotation; Figure S3: Kyoto Encyclopedia of Genes and Genomes (KEGG) pathway annotation; Figure S4: Antifungal activity spectrum of the strain YBS22; Figure S5: Examining the antifungal activity of various components within the crude extract. Figure S6: Flow chart of isolation and purification of antibacterial activity product from the crude extract of the strain YBS22. Figure S7: HPLC of compound **1**. Figure S8: Mass spectrum of compound **1**. Figure S9: The ^1^H and ^13^C NMR spectra of compound **1**. Table S1: Basic information of YBS22 genome; Table S2: GO Annotation Data; Table S3: KEGG Annotation Data; Table S4: Predicted results of antibiotics secondary metabolite; Table S5: Effect of pH and heat stability on crude extract of the strain YBS22 against *M. oryzae*. Table S6: Weight and antifungal activity of components **T1**~**T8**. Table S7: ^1^H and ^13^C NMR Assignments for Compound **1**.
