# Supplementary material for: Isolation and Evaluation of Streptomyces melanogenes YBS22 with Potential Application for Biocontrol of Rice Blast Disease
Source: Microorganisms. 2023 Dec 15;11(12):2988. doi: 10.3390/microorganisms11122988 (PMC10745888; doi:10.3390/microorganisms11122988)
Supplement: Supplementary file 1 [file microorganisms-11-02988-s001.zip › Suppl-FigureS1_S9-2.pdf]

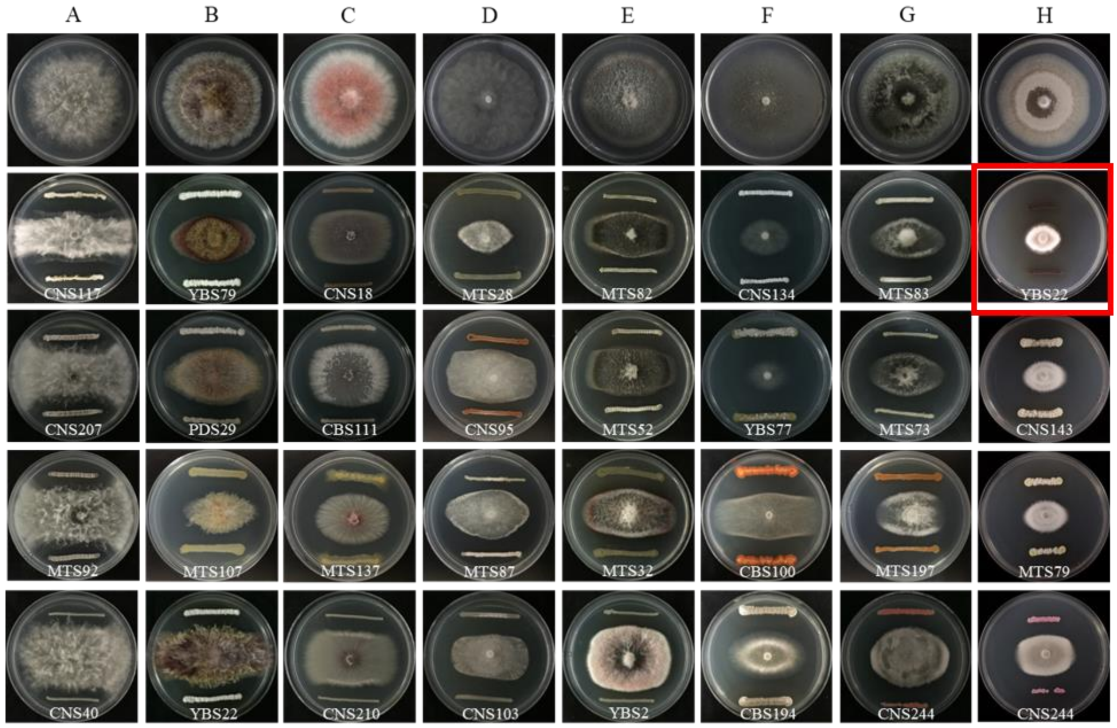

Figure S1. Antagonistic activity profiles of typical strains. Note: A-H represents A; *B. dothidea*, B; *F. graminearum*, C; *F. oxysporum* f. sp. *momodicase*, D; *P. capsici*, E; *C. orbiculare*, F; *G. graminis* var. *tritici*, G; *H. turcicum*, H; *M. oryzae*, respectively.

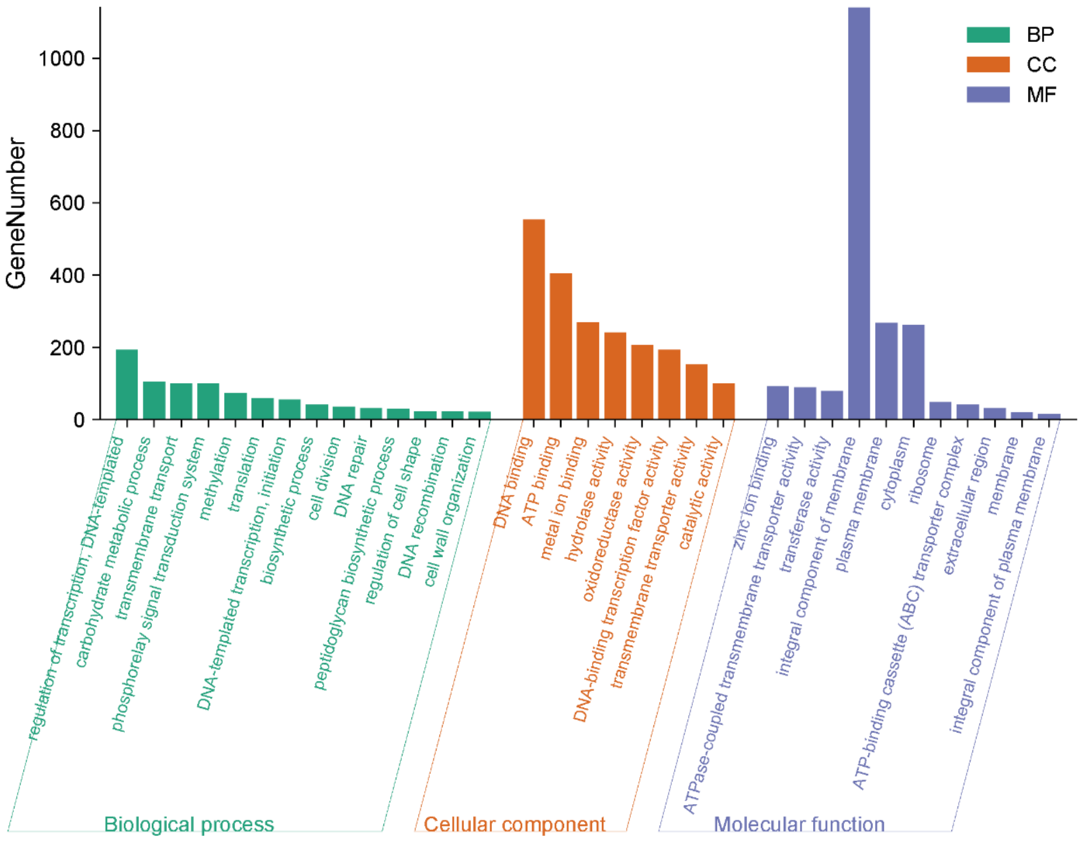

Figure S2. GO annotation. Note: A total of 4,865 genes were classified into 30 functional groups, and the genes involved in molecular function were most abundantly.

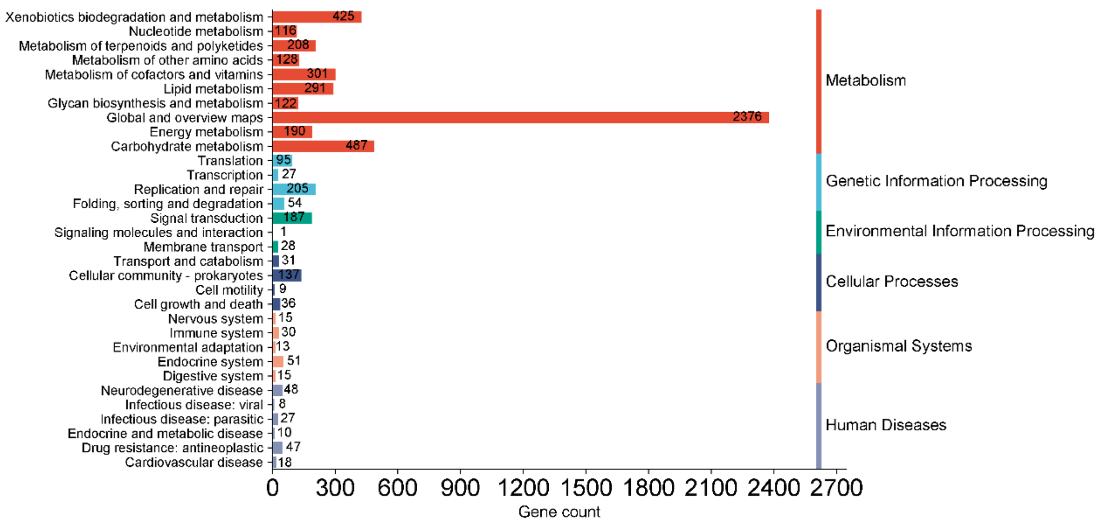

Figure S3. Kyoto Encyclopedia of Genes and Genomes (KEGG) Pathway annotation. 5850 genes (73.7% of all CDSs.) were assigned to 32 KEGG pathways, and the largest number of identified genes were classified into metabolism pathways.

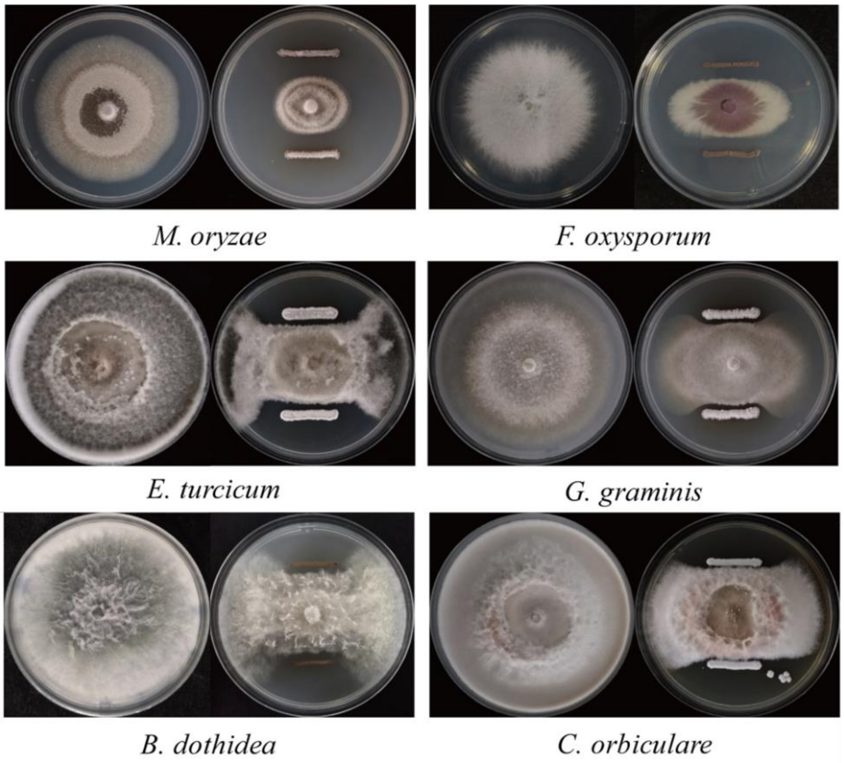

Figure S4. Antifungal Activity Spectrum of Strain YBS22.

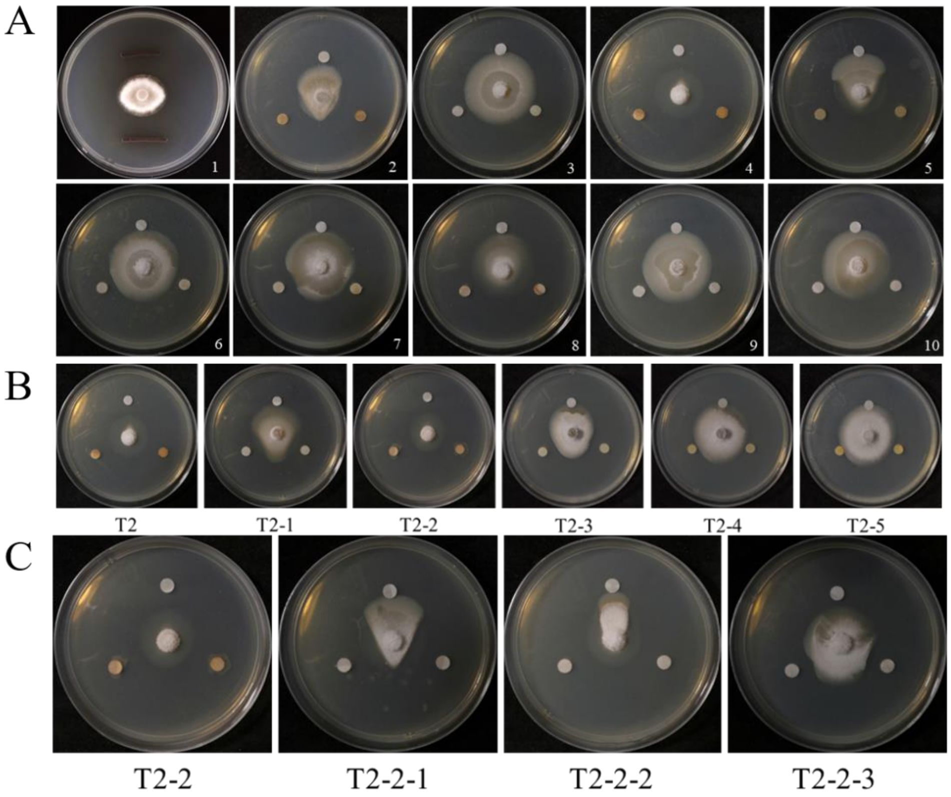

Figure S5. Examining the antifungal activity of various components within the crude extract. A; Antifungal activity of components T1~T8 against *M. oryzae*. B; Antifungal activity of components T2-1~T2-5 against M. oryzae. C; Antifungal activity of components T2-2-1~T2-2-3 against *M. oryzae*.

Note; 1: dual culture control group; 2: Methanol solution of crude extract from solid fermentation; 3-10: Components T1, T2, T3, T4, T5, T6, T7, and T8 in order.

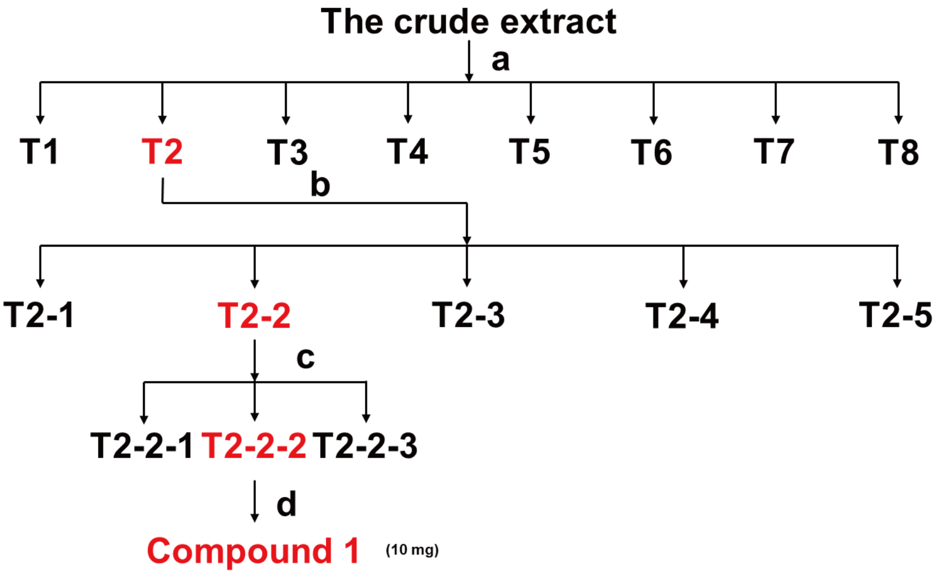

Figure S6. Flow chart of isolation and purification of antibacterial activity product from the crude extract of strain YBS22. Note; a: First-class silica gel column chromatography separation; b: Secondary silica gel column chromatography separation; c: Sephadex LH-20 column

chromatography; d: Recycling preparative HPLC.

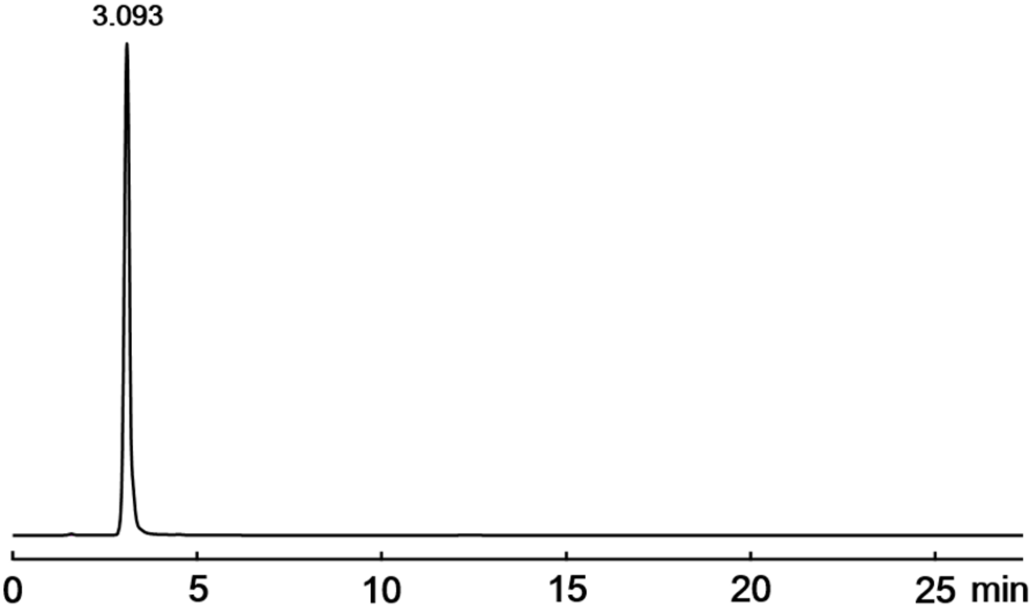

Figure S7. HPLC of compound **1**. HPLC (TC-C18 column, 4.6×250 mm, 5 µm) chromatographic spectrum of the yellow amorphous solid, which displayed a single peak at about 3.09 min and marked as compound **1**.

Elmt	Val.	Min	Max	Elmt	Val.	Min	Max	Elmt	Val.	Min	Max	Elmt	Val.	Min	Max	Use Adduct
H	1	0	50	N	3	0	5	Na	1	0	0	Cl	1	0	0	Na
B	3	0	0	O	2	0	10	P	3	0	0	Br	1	0	0	
C	4	10	30	F	1	0	0	S	2	0	0					

Error Margin (ppm):	9999	DBE Range:	not fixed	Electron Ions:	both
HC Ratio:	0.0 - 100.0	Apply N Rule:	yes	Use MSn Info:	no
Max Isotopes:	all	Isotope RI (%):	1.00	Isotope Res:	10000
MSn Iso RI (%):	75.00	MSn Logic Mode:	AND	Max Results:	50

Event#: 1 MS(E+) Ret. Time : 10.033 Scan# : 1027

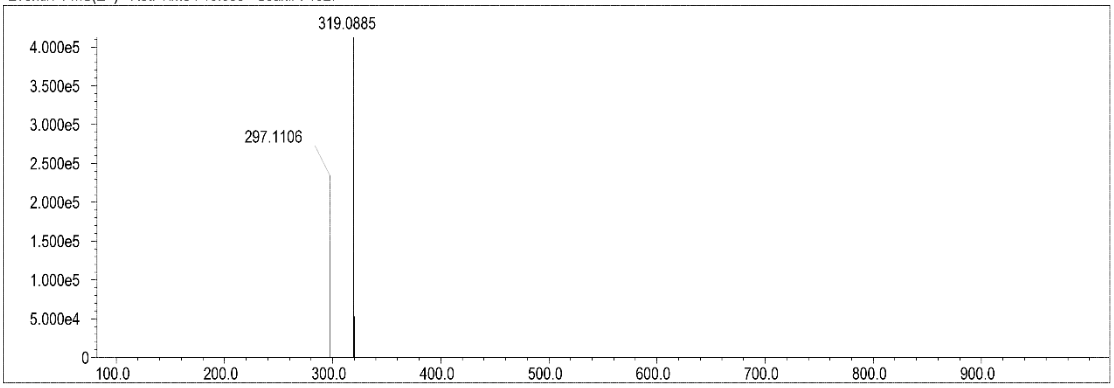

Measured region for 319.0885 m/z

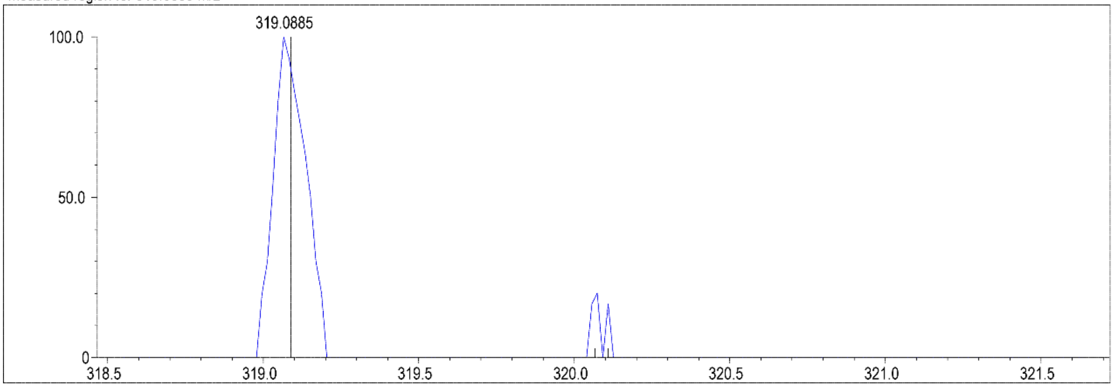

C13 H16 N2 O6 [M+Na]+ : Predicted region for 319.0901 m/z

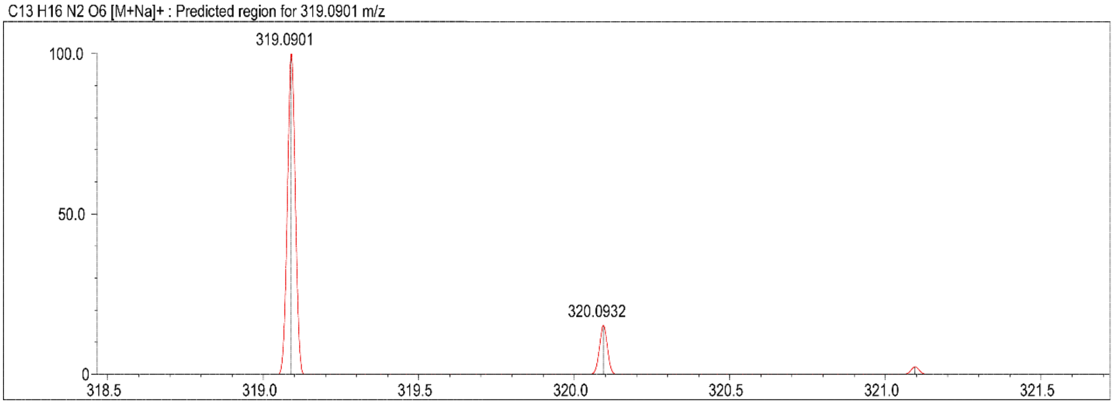

Rank	Score	Formula (M)	Ion	Meas. m/z	Pred. m/z	Df. (mDa)	Df. (ppm)	Iso	DBE
1	5.15	C13 H16 N2 O6	[M+Na]+	319.0885	319.0901	-1.6	-5.01	5.72	7.0

Figure S8. Mass spectrum of compound **1**. The molecular formula of compound **1** was subsequently confirmed as $C_{13}H_{16}O_6N_2$ by positive-ion high-resolution electrospray ionization mass spectrometry (HRESIMS)

A

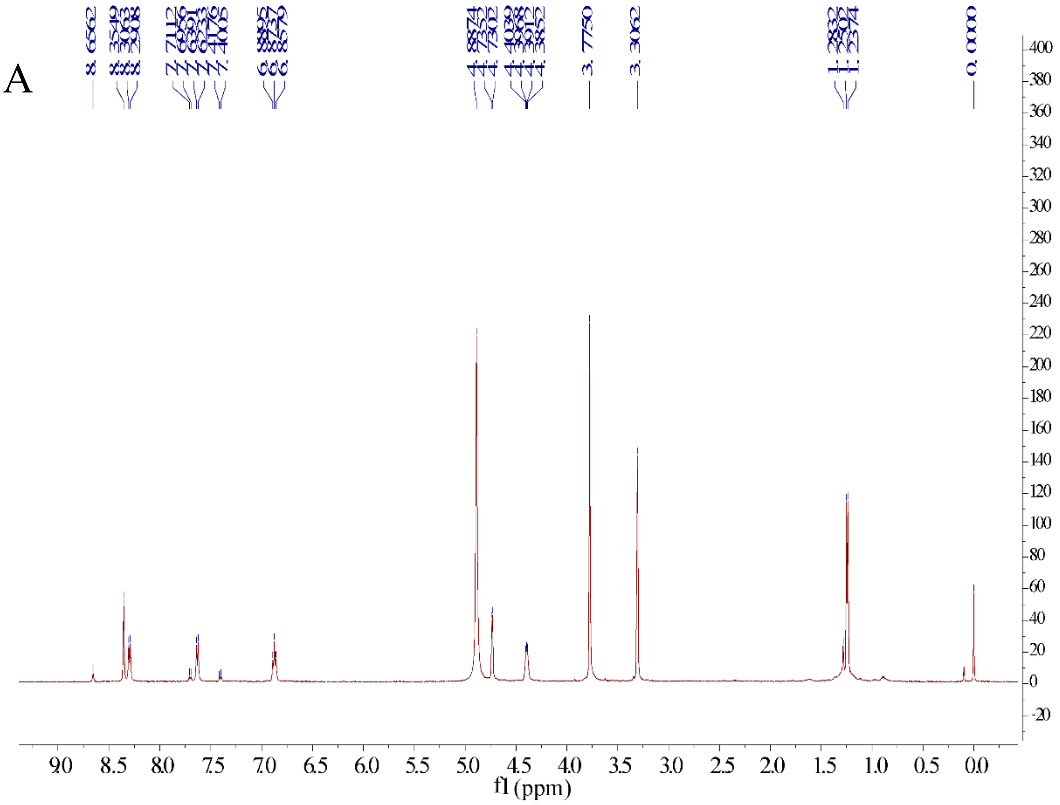
8.6562
8.3549
8.3363
8.3308
7.7112
7.6656
7.6591
7.6233
7.4176
7.4005
6.8895
6.8737
6.8579
4.8874
4.7355
4.7302
4.4039
4.3968
4.3912
4.3852
3.7750
3.3062
1.2832
1.2502
1.2374
0.0000
f1 (ppm)

B

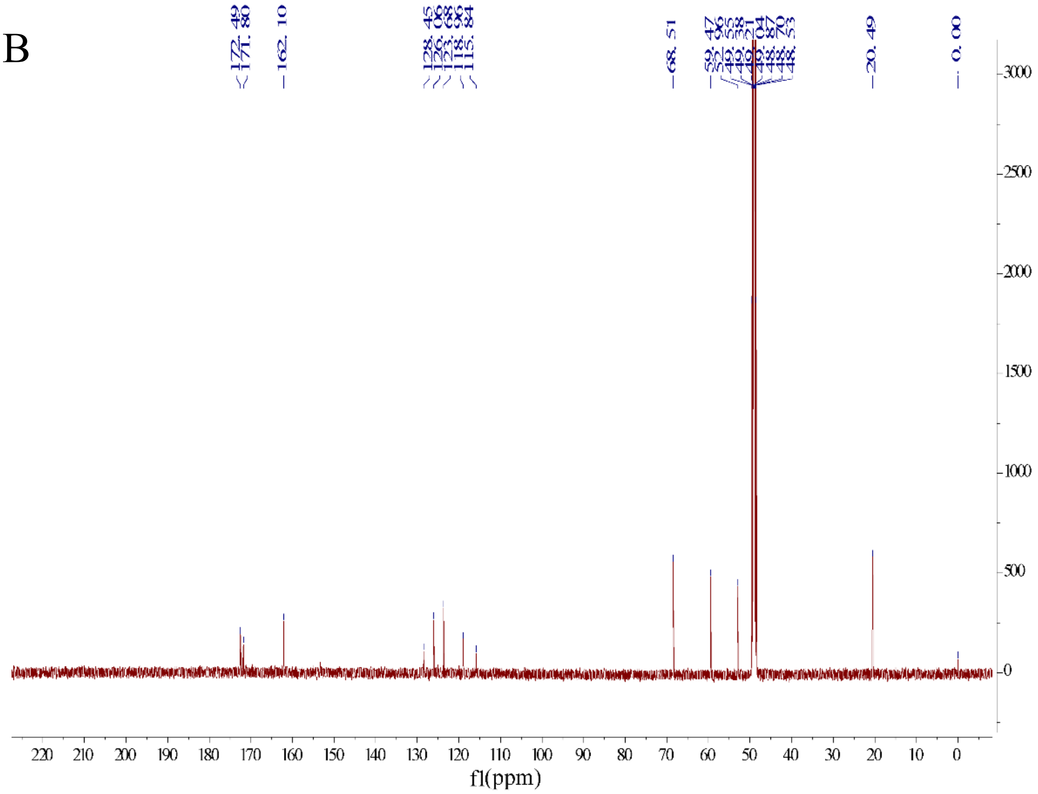
172.49
171.80
162.10
128.45
126.06
123.68
118.96
115.84
68.51
59.47
52.96
49.55
49.38
49.21
49.04
48.87
48.70
48.53
20.49
0.00
f1(ppm)

C

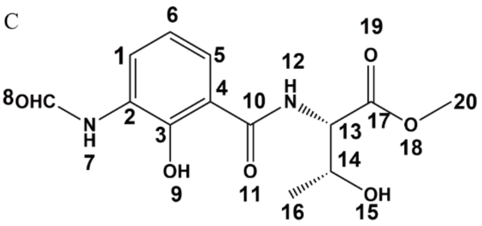

Figure S9. The ^{1}H and ^{13}C NMR spectrums of compound **1**. A; H NMR chart of compound **1** (in Methanol-d_4). B; ^{13}C NMR chart of compound **1** (in Methanol-d_4). C; Structure of the compound **1** (*N*-formylantimycic acid methyl ester).
